# Identification of metabolism terms significantly affecting hepatocellular carcinoma immune microenvironment and immunotherapy response

**DOI:** 10.1111/jcmm.18018

**Published:** 2023-11-09

**Authors:** Xijuan Tan, Sizong Chen, Qiyi Luo, Shenglin You, Hankun Yuan, Jianchu Wang

**Affiliations:** ^1^ Department of Hepatobiliary Surgery Affiliated Hospital of Youjiang Medical University for Nationalities Guangxi China

**Keywords:** immune, immunotherapy, liver cancer, metabolism, signature

## Abstract

Metabolic pathways exert a significant influence on the onset and progression of cancer. Public data on hepatocellular carcinoma (HCC) patients were obtained from The Cancer Genome Atlas (TCGA) and International Cancer Genome Consortium (ICGC) databases. Analysis was performed in R software using different R packages. Here, we integrated the data from multiple independent HCC cohorts, including TCGA‐LIHC, ICGC‐FR and ICGC‐JP. Then, the enrichment score of 21 metabolism‐related pathways was quantified using the ssGSEA algorithm. Next, univariate Cox regression analysis was applied to identify the metabolic terms with significant correlation to patient survival. Finally, a prognosis model based on linoleic acid metabolism, sphingolipid metabolism and regulation of lipolysis in adipocytes was established, which showed good performance in predicting patients' survival. Furthermore, we conducted a biological enrichment analysis to delineate the biological disparities between high‐ and low‐risk patients. Notably, we discerned differences in the microenvironments between these two patient groups. We also found that low‐risk patients could potentially respond better to immunotherapy. Drug sensitivity analysis suggested that low‐risk patients are more susceptible to bexarotene and erlotinib, yet exhibit resistance to ATRA and bleomycin. Furthermore, through the use of LASSO logistic regression analysis, we identified 19 characteristic genes, which could robustly indicate the risk groups. Our research underscores the role of linoleic acid metabolism, sphingolipid metabolism and the regulation of lipolysis in adipocytes in HCC, pointing towards potential avenues for future research.

## 1 INTRODUCTION

Liver cancer, one of the most prevalent malignancies globally, exhibits especially high incidence and mortality rates.^1^ Hepatocellular carcinoma (HCC), the dominant pathological subtype of liver cancer, largely stems from hepatitis virus infections, prolonged alcohol consumption, exposure to aflatoxins and metabolic disorders.^2^, ^3^ Most patients with HCC are unfortunately diagnosed at an advanced stage, which generally results in a grim prognosis and a comparatively low 5‐year survival rate.^4^, ^5^ The primary therapeutic strategies for HCC presently encompass surgical excision, liver transplantation, localized treatment and systemic therapy.^6^ However, due to the intricacy of the disease and the hurdles associated with late‐stage diagnosis, these treatments often fail to deliver satisfactory outcomes.^7^ In this context, the identification of reliable biomarkers carries immense importance for the early detection and tailored treatment of liver cancer.^8^ Through the assessment of specific biomarkers, liver cancer can potentially be diagnosed at an earlier stage, enabling the development of more accurate treatment strategies. This can enhance patients' quality of life and prognostic outlook. Moreover, the rigorous investigation into biomarkers might unveil novel therapeutic targets, paving the way for the creation of more efficacious drug therapies.^9^

Metabolic reprogramming is now recognized as a hallmark of cancer, involving key changes in glucose, lipid and amino acid metabolism.^10^ In HCC, these alterations not only fuel rapid tumour growth but also facilitate the evasion of immune responses and promote drug resistance, thereby contributing to the aggressive nature of this malignancy.^11^ Several studies have demonstrated the involvement of key metabolic pathways in HCC. For example, a comprehensive review from Vander Heiden et al. mentioned the altered glucose metabolism in HCC, uncovering the key role of glycolysis—an energy production pathway that is often upregulated in HCC. They showed that HCC cells favour glycolysis, even in the presence of oxygen, a phenomenon known as the Warburg effect.^12^ This supports rapid energy production and biomass generation, facilitating tumour growth.^13^ Seo et al. discovered that the fatty acid‐induced FABP5/HIF‐1 axis can modify lipid metabolism and enhance the proliferation of hepatic cancer cells.^14^ In a related study, Xu and his team uncovered that hepatic sulphur amino acid (SAA) metabolism undergoes transcriptional modulation under the influence of HNF4α. This modulation could potentially affect the sensitivity of liver cancer to methionine restriction.^15^ These findings underscore the imperative need to investigate potential clinical targets for HCC from a metabolic standpoint.

In this study, we integrated the data from multiple independent HCC cohorts, including TCGA‐LIHC, ICGC‐FR and ICGC‐JP. Then, the enrichment score of 21 metabolism‐related pathways was quantified using the ssGSEA algorithm. Next, univariate Cox regression analysis was applied to identify the metabolic terms with significant correlation to patient survival. Finally, a prognosis model based on linoleic acid metabolism, sphingolipid metabolism and regulation of lipolysis in adipocytes was established, which showed good performance in predicting patient survival. Furthermore, we conducted a biological enrichment analysis to delineate the biological disparities between high‐ and low‐risk patients. Notably, we discerned differences in the microenvironments between these two patient groups. We also found that low‐risk patients could potentially respond better to immunotherapy. Drug sensitivity analysis suggested that low‐risk patients are more susceptible to bexarotene and erlotinib, yet exhibit resistance to ATRA and bleomycin. Furthermore, through the use of LASSO logistic regression analysis, we identified 19 characteristic genes, which could robustly indicate the risk groups.

## 2 METHODS

### 2.1 Open‐accessed data and processing

The transcriptomic and clinical details of TCGA‐LIHC (Liver Hepatocellular Carcinoma) were directly retrieved from the Genomic Data Commons (GDC) Data Portal.^16^ The ICGC‐JP and ICGC‐FR cohorts were downloaded from the ICGC Data Portal. After downloading, data preprocessing was performed to ensure quality analysis, including normalization of gene expression data, dealing with missing values and annotating the data with gene symbols. The Sva R package was used for data merging and bias effect removal. Differentially expressed gene analysis was conducted using the limma package.^17^

### 2.2 Single‐sample gene set enrichment analysis

Single‐sample gene set enrichment analysis (ssGSEA) ranks genes based on their absolute expression levels and then uses these rankings to calculate an enrichment score.^18^ This score is then normalized, taking into account the size of the gene set, to generate the final ssGSEA score. Based on a specific metabolism gene set, we quantified the score of 21 metabolism‐related pathways.

### 2.3 Prognosis analysis and model construction

Firstly, the merged cohort is randomly divided into a training set and a validation set in a 1:1 ratio. Prognostic model construction involved a three‐step process: (1) Univariate Cox regression analysis; (2) LASSO regression analysis; (3) Multivariate Cox regression analysis. Univariate Cox regression analysis is the initial screening step to identify potential prognostic factors. By examining the relationship between each factor and the survival outcome separately, factors that have a significant impact on survival (*p* < 0.05) are identified. LASSO regression is a type of regularization method used to avoid overfitting and to perform feature selection. It shrinks the coefficients of less important features to zero, effectively selecting a subset of features for the next step. Multivariate Cox regression analysis takes the subset of features identified by LASSO and evaluates their collective impact on survival outcome, adjusting for potential confounding variables. The final prognostic model is based on the significant predictors from this analysis. To gauge the effectiveness of the model, Kaplan–Meier (KM) survival curves were employed to juxtapose the survival outcomes of various risk groups designated by the model. The log‐rank test frequently served to assess the statistical significance of the observed differences. In addition, receiver operating characteristic (ROC) curves were leveraged to appraise the predictive precision of the model, where an AUC value of 1 connotes flawless accuracy, and an AUC of 0.5 suggests a predictive power equivalent to a random guess. To improve the clinical application potential of the constructed model, we merged the risk score and clinical features to construct the nomogram.

### 2.4 Biological enrichment analysis

Biological enrichment was conducted using GSEA algorithm based on specific gene sets.^19^

### 2.5 Tumour microenvironment analysis

The CIBERSORT algorithm was utilized to quantify the level of immune cell infiltration in tumour tissues.^20^ The Estimate package was employed to deduce the proportion of stromal and immune cells in tumour specimens.

### 2.6 Immunotherapy and drug sensitivity analysis

Tumour immune dysfunction and exclusion (TIDE) analysis uses expression profiles of tumours to forecast the response to immune checkpoint blockade.^21^ The Genomics of Drug Sensitivity in Cancer (GDSC) database is a comprehensive resource providing information about the sensitivity of cancer cells to anticancer drugs.^22^

### 2.7 Feature variable screening

The screening of feature variables was conducted using the LASSO logistic regression, which is a statistical method that combines logistic regression with the LASSO penalty.

### 2.8 Statistical analysis

All statistical analyses were undertaken using R software (version 3.6.1). Descriptive statistics were calculated for all study variables. A two‐sided *p*‐value of less than 0.05 was deemed statistically significant in all analyses.

## 3 RESULTS

### 3.1 Quantification of enrichment score of metabolism‐related pathways

**FIGURE 1:**
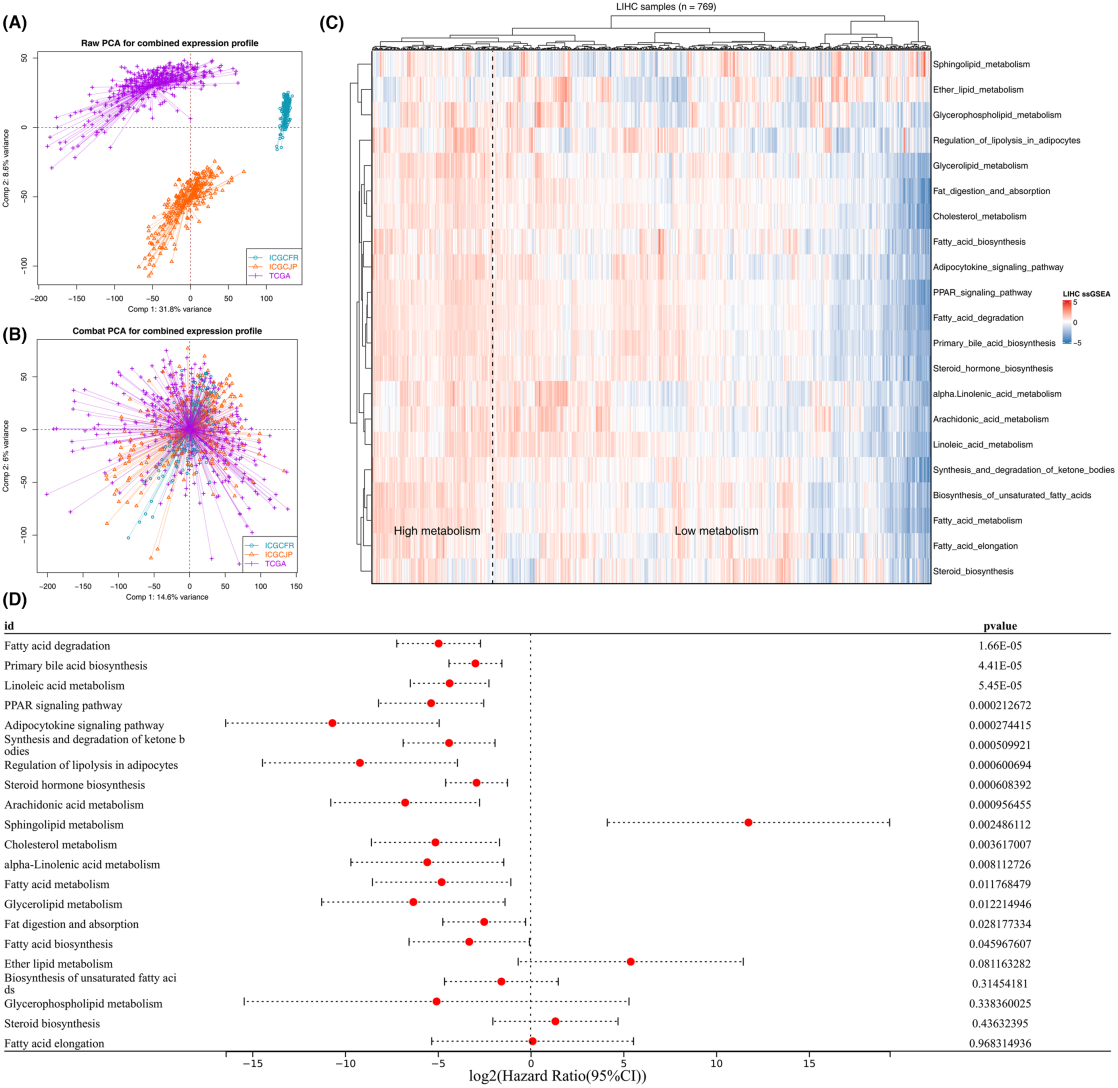
Quantification of metabolism term in HCC. (A, B) Sva package was used to reduce the batch effect of TCGA‐LIHC, ICGC‐JP, and ICGC‐FR. (C) The enrichment score of 21 metabolism pathways was quantified using the ssGSEA algorithm. (D) Univariate Cox regression analysis was used to identify the metabolism terms significantly correlated with patients survival.

Initially, we gathered transcriptome data from three cohorts: TCGA‐LIHC, ICGC‐JP and ICGC‐FR. A notable batch effect was present between these cohorts (Figure 1A, with Comp 1 accounting for 31.8% of variance and Comp 2 for 8.6%). After implementing the Sva package for data consolidation and batch effect mitigation, it became evident that the three cohorts could be effectively integrated (Figure 1B, Comp 1: 14.6% variance; Comp 2: 6% variance). Subsequently, we quantified the enrichment scores for 21 metabolism‐related pathways, based on the transcriptome data from the merged cohorts (Figure 1C). Finally, to identify metabolism terms with a significant correlation to patient prognosis, we performed a univariate Cox regression analysis (Figure 1D, *p* < 0.05).

### 3.2 A prognosis model based on metabolism terms

**FIGURE 2:**
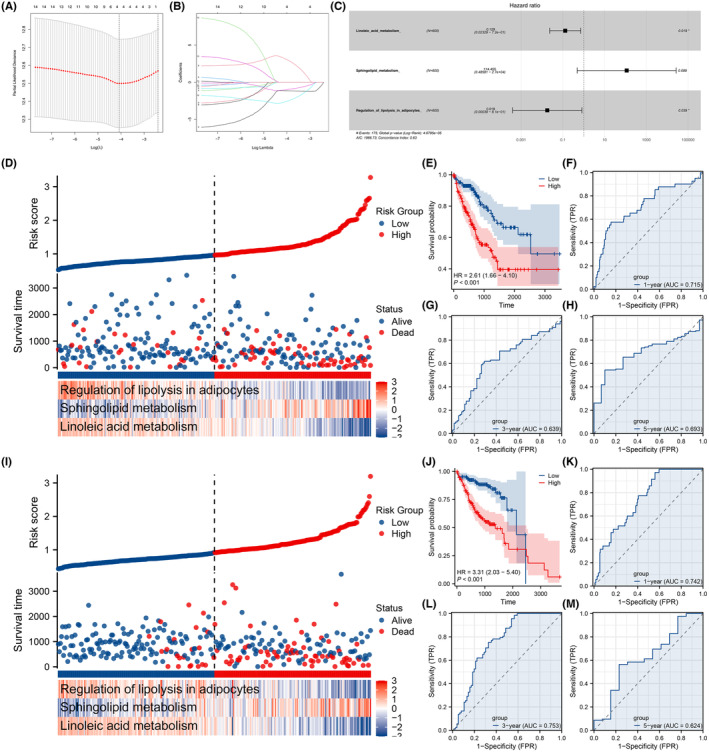
Prognosis model construction. (A, B) LASSO regression algorithm was used to reduce data dimensions and optimize variables. (C) Multivariate Cox regression analysis was utilized to screen the final variables for prognosis model. (D) Overview of prognosis model in the training cohort. (E) KM survival curves of high‐ and low‐risk patients (training cohort). (F–H) ROC curves of risk score in the training cohort (1, 3 and 5 years). (I) Overview of prognosis model in the validation cohort. (J) KM survival curves of high‐ and low‐risk patients (validation cohort). (K–M) ROC curves of risk score in the validation cohort (1, 3 and 5 years).

Building upon the variables identified by the univariate Cox regression analysis, we employed LASSO regression analysis for further dimension reduction (Figure 2A,B). A subsequent multivariate Cox regression analysis highlighted three metabolic terms for the construction of the prognosis model: linoleic acid metabolism, sphingolipid metabolism and regulation of lipolysis in adipocytes (Figure 2C). The risk score was computed using the formula: ‘Risk score = (linoleic acid metabolism × −2.045) + (sphingolipid metabolism × 4.740) + (regulation of lipolysis in adipocytes × −4.026)’. In the training cohort, we observed a higher incidence of mortality in the high‐risk group (Figure 2D). The KM survival curves suggested a potentially poorer prognosis for high‐risk patients (Figure 2E, HR = 2.61, *p* < 0.001). The ROC curves demonstrated that our prognosis model performed well in predicting patient survival (Figure 2F–H, with 1‐year AUC = 0.715, 3‐year AUC = 0.639, 5‐year AUC = 0.693). These conclusions were further reinforced in the validation cohort (Figure 2I–M, HR = 3.31, *p* < 0.001; 1‐year AUC = 0.742, 3‐year AUC = 0.753, 5‐year AUC = 0.624). Moreover, a nomogram plot combining the risk score and clinical features was constructed (Figure S1).

### 3.3 Clinical correlation of linoleic acid metabolism, sphingolipid metabolism and regulation of lipolysis in adipocytes

**FIGURE 3:**
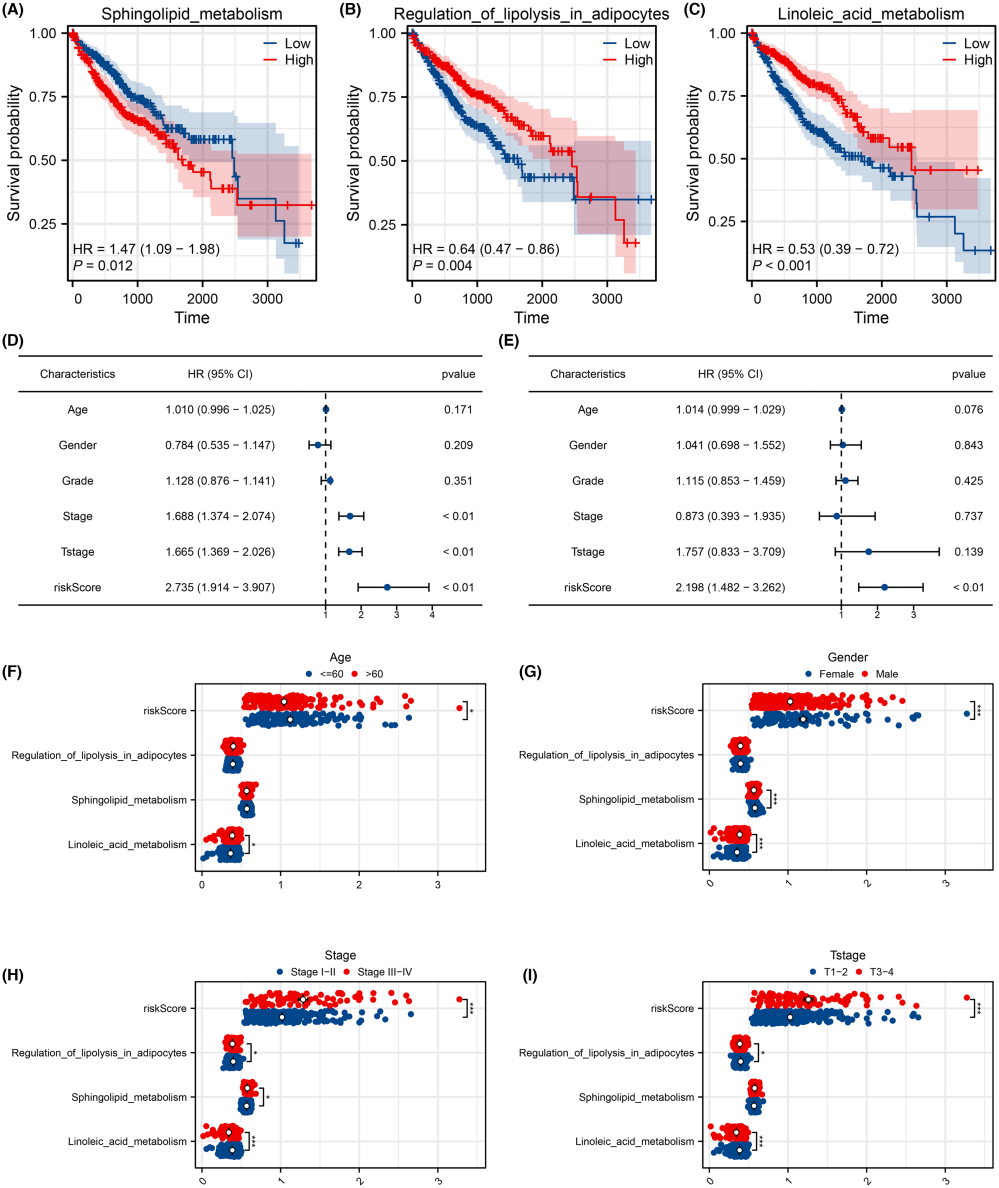
Clinical role of model metabolism terms. (A) KM survival curves of patients with high and low sphingolipid metabolism level. (B) KM survival curves of patients with high and low regulation of lipolysis in adipocytes level. (C) KM survival curves of patients with high and low linoleic acid metabolism level. (D) Univariate analysis of risk score and clinical features. (E) Multivariate analysis of risk score and clinical features. (F–I) The level of risk score and model metabolism terms in patients with different clinical features. *p < 0.05; ***p < 0.001.

KM survival curves demonstrated that patients with elevated sphingolipid metabolism levels tend to have a poorer survival outcome (Figure 3A, HR = 1.47, *p* < 0.012). Conversely, patients with higher levels of linoleic acid metabolism and regulation of lipolysis in adipocytes exhibited a more favourable survival performance (Figure 3B,C, linoleic acid metabolism, HR = 0.53, *p* < 0.001; regulation of lipolysis in adipocytes, HR = 0.64, *p* = 0.004). Subsequent univariate and multivariate analyses revealed that the risk score serves as an independent prognostic marker for HCC patients (Figure 3D, univariate analysis, HR = 2.735, *p* < 0.01; Figure 3E, multivariate analysis, HR = 2.198, *p* < 0.01). Clinical correlation analysis indicated that the risk score was elevated in patients under 60 years old (Figure 3F); both the risk score and sphingolipid metabolism were higher, whereas linoleic acid metabolism was lower in female patients (Figure 3G); the risk score and sphingolipid metabolism were amplified, yet the regulation of lipolysis in adipocytes and linoleic acid metabolism were diminished in stage III–IV patients (Figure 3H); the risk score was increased, while regulation of lipolysis in adipocytes and linoleic acid metabolism was reduced in T3–T4 patients (Figure 3I).

### 3.4 Biological enrichment analysis

**FIGURE 4:**
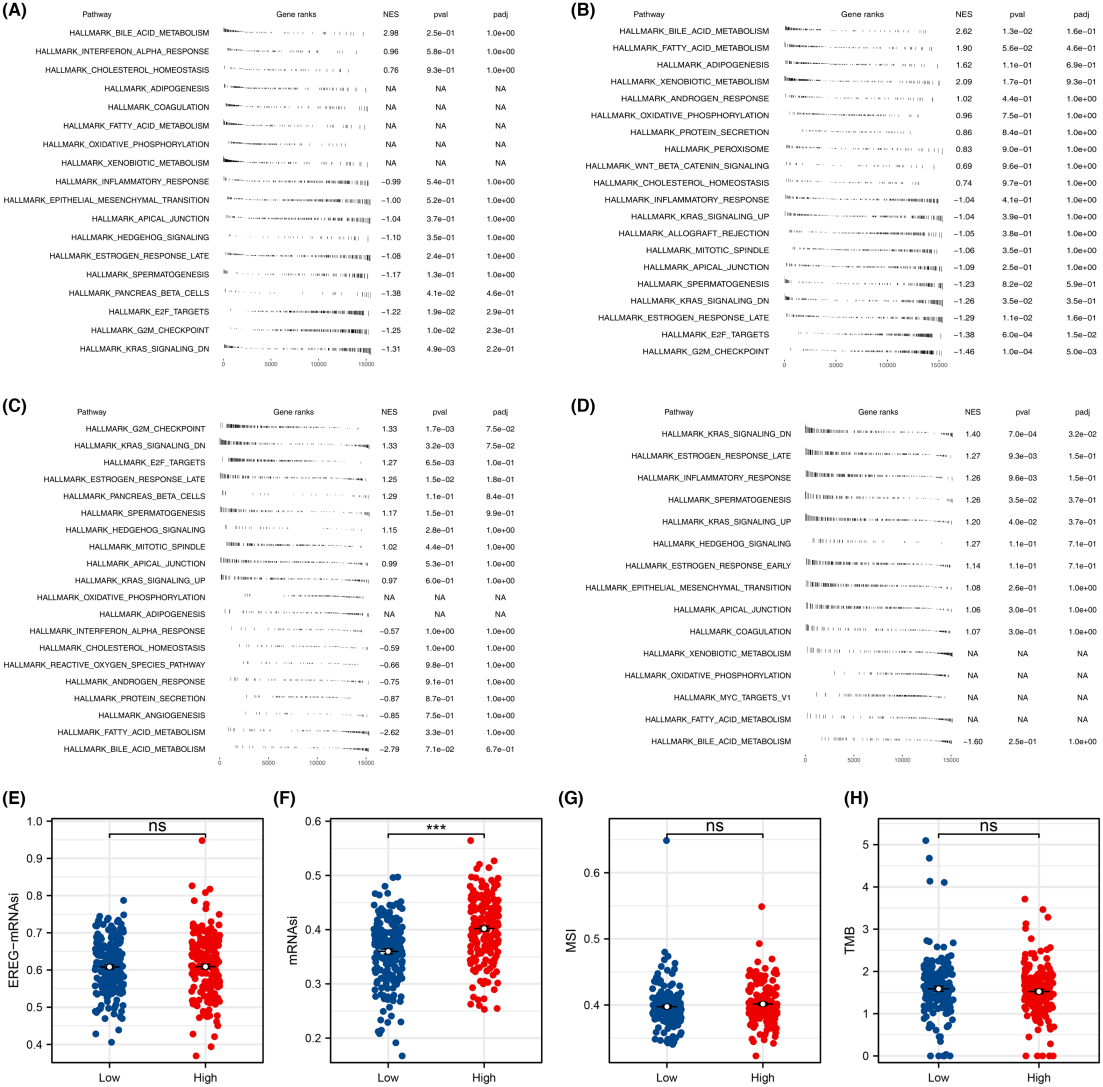
Biological enrichment analysis. (A) GSEA analysis based on Hallmark gene set of linoleic acid metabolism. (B) GSEA analysis based on Hallmark gene set of regulation of lipolysis in adipocytes. (C) GSEA analysis based on Hallmark gene set of risk score. (D) GSEA analysis based on Hallmark gene set of sphingolipid metabolism. (E, F) The EREG‐mRNAsi and mRNAsi level in high‐ and low‐risk patients, ^ns^ *p* > 0.05, ***p < 0.001. (G, H) The TMB and MSI level in high‐ and low‐risk patients, ^ns^ *p* < 0.05.

Subsequently, we sought to investigate the biological impact of the model's metabolic terms and risk score. GSEA analysis, using the Hallmark gene set, suggested that linoleic acid metabolism primarily contributes to bile acid metabolism, interferon alpha response and cholesterol homeostasis (Figure 4A). The regulation of lipolysis in adipocytes predominantly participates in bile acid metabolism, fatty acid metabolism and adipogenesis (Figure 4B). The risk score is principally involved in G2M checkpoints, KRAS signalling and E2F targets (Figure 4C). Sphingolipid metabolism is primarily associated with KRAS signalling, late oestrogen response and inflammatory response (Figure 4D). Furthermore, we found that the risk score was linked to elevated tumour stemness (Figure 4E,F), but not genomic instability (Figure 4G,H).

### 3.5 Immune microenvironment analysis

**FIGURE 5:**
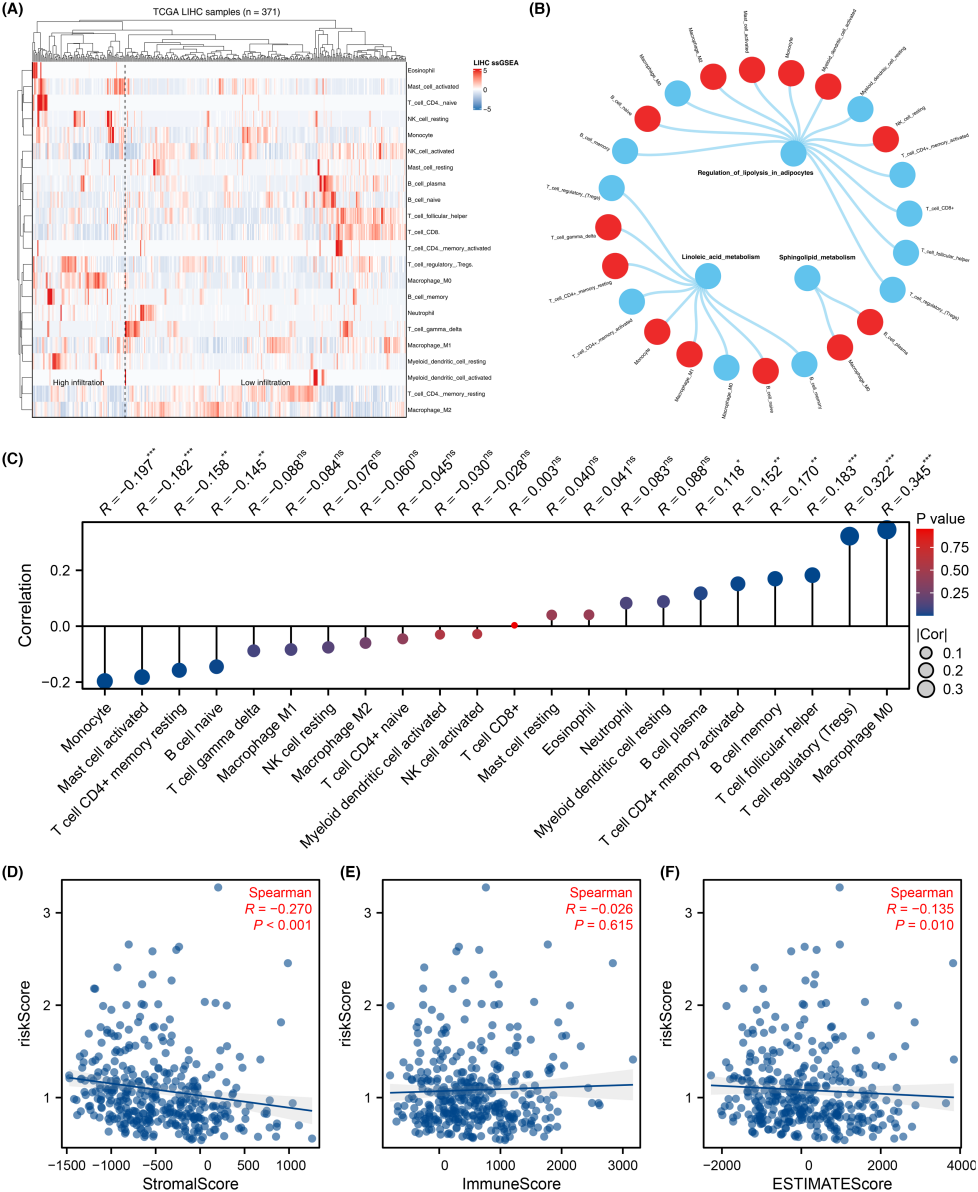
Immune microenvironment analysis. (A) CIBERSORT algorithm was used to quantify the immune microenvironment of HCC tissue. (B) Correlation between linoleic acid metabolism, regulation of lipolysis in adipocytes, sphingolipid metabolism and immune cell. (C) Correlation between risk score and immune cells, ^ns^ *p* > 0.05, *p < 0.05, **p < 0.01, ***p < 0.001. (D) Correlation between risk score and stromal score. (E) Correlation between risk score and immune score. (F) Correlation between risk score and estimate score.

We employed the CIBERSORT algorithm to quantify the immune microenvironment of HCC tissue sourced from the TCGA‐LIHC database (Figure 5A). The correlation analysis indicated that linoleic acid metabolism has a positive correlation with naive B cells, gamma delta T cells, resting memory CD4+ T cells, monocytes and M1 macrophages, but exhibits a negative correlation with Tregs, memory B cells, activated memory CD4+ T cells and M0 macrophages. Sphingolipid metabolism positively correlates with M0 macrophages and plasma B cells. Regulation of lipolysis in adipocytes has a positive association with resting NK cells, activated myeloid dendritic cells, monocytes, activated mast cells and M2 macrophages, but negatively correlates with Tregs and CD8+ T cells (Figure 5B). Concurrently, the risk score was positively correlated with M0 macrophages, Tregs and follicular helper T cells, but exhibits a negative correlation with monocytes (Figure 5C). In addition, the risk score was observed to have a negative correlation with the stromal score and estimate score, but not with the immune score (Figure 5D–F).

### 3.6 Difference between immunotherapy and drug sensitivity analysis

**FIGURE 6:**
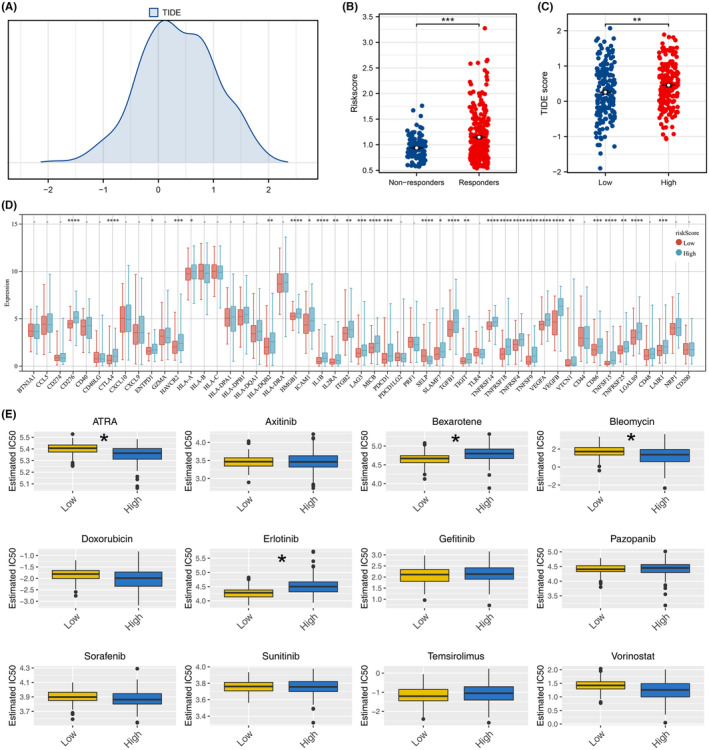
Immunotherapy and drug sensitivity difference between high‐ and low‐risk patients. (A) Distribution of TIDE score quantified by TIDE analysis. (B) Level of risk score in immunotherapy responders and non‐responders, ***p < 0.001. (C) Level of TIDE score in high‐ and low‐risk patients. **p < 0.01. (D) Level of immune checkpoints in high‐ and low‐risk patients, *p < 0.05, **p < 0.01, ***p < 0.001, ****p < 0.0001. (E) IC50 of specific drugs in high‐ and low‐risk patients, *p < 0.05.

Subsequently, we calculated the TIDE score using TIDE analysis to elucidate the disparity in immunotherapy responses (Figure 6A). The findings showed that immune responders have a lower risk score and high‐risk patients are inclined to exhibit a higher TIDE score. This suggests that low‐risk patients may exhibit a better response to immunotherapy (Figure 6B,C). Moreover, we discovered that most immune checkpoints showed differential expression between high‐ and low‐risk patients (Figure 6D). The results from the drug sensitivity analysis indicated that low‐risk patients are more responsive to bexarotene and erlotinib, yet display resistance to ATRA and bleomycin (Figure 6E).

### 3.7 Identification of the characteristic genes

**FIGURE 7:**
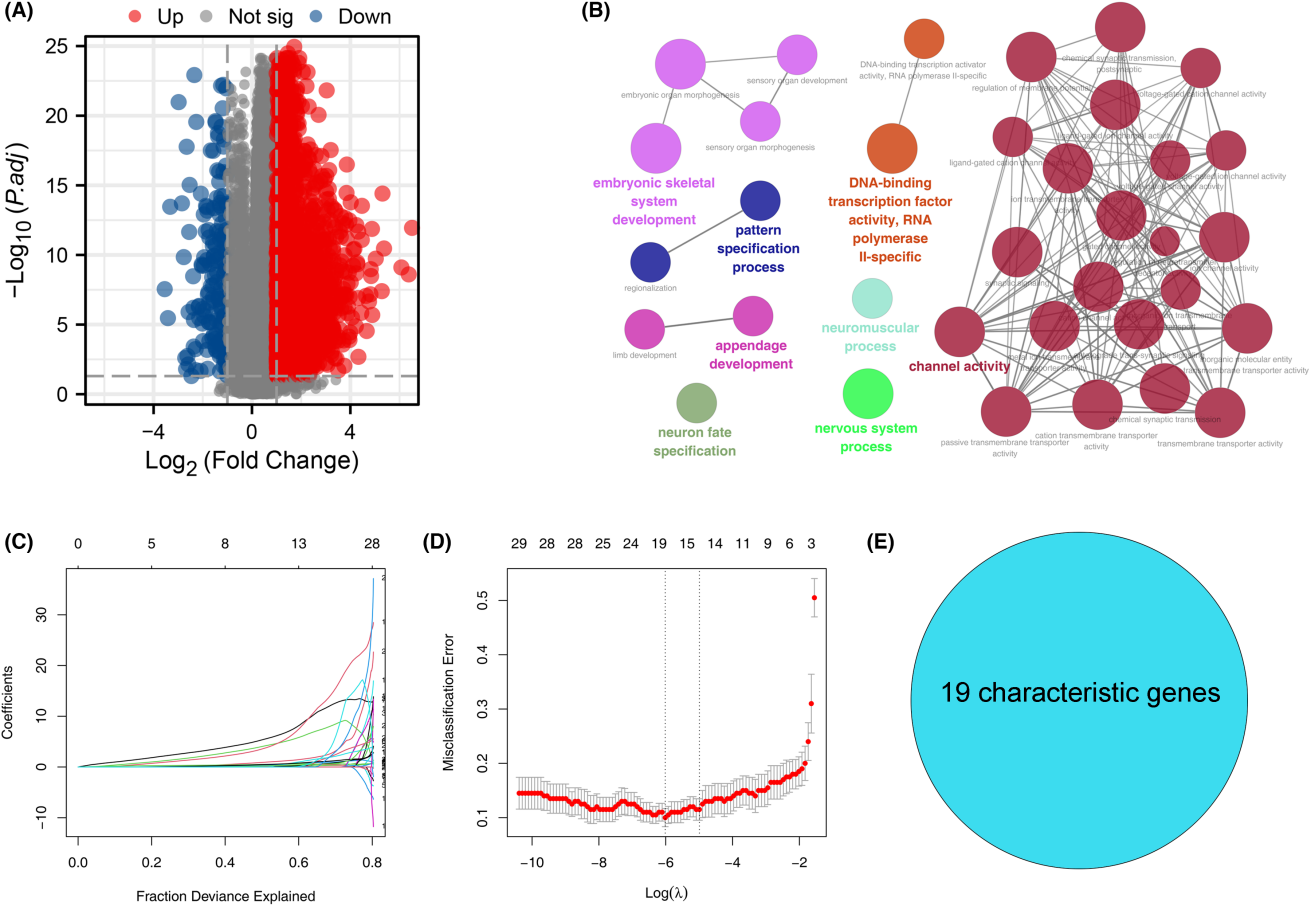
Identification of the characteristic gene of risk group. (A) DEG analysis was performed between high‐ and low‐risk patients. (B) ClueGO analysis of DEGs. (C–E) LASSO logistic regression was utilized to identify the characteristic gene of risk group.

**FIGURE 8:**
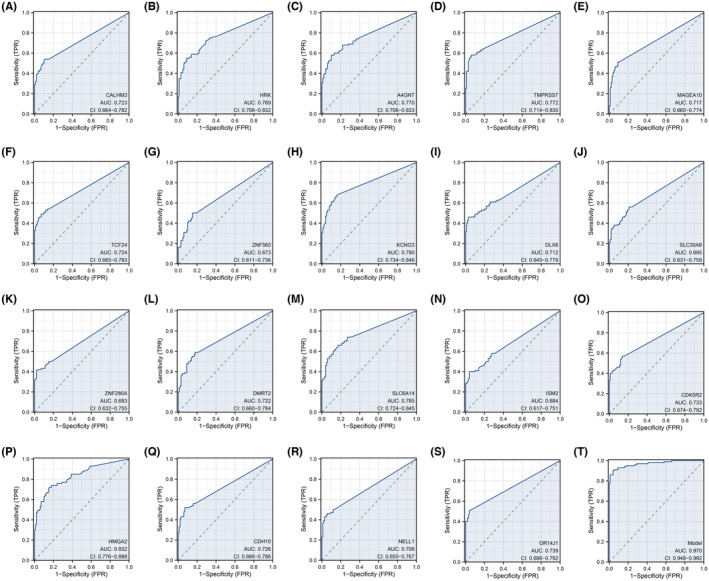
Performance of identified characteristic genes. (A–S) Prediction efficiency of identified characteristic genes in predicting risk group. (T) Prediction efficiency of model score in predicting risk group.

To pinpoint the defining genes of the risk score and to better delineate high and low‐risk groups, we carried out a DEG analysis between high and low‐risk patients (Figure 7A). The ClueGO analysis revealed that these DEGs were predominantly involved in processes such as embryonic skeletal system development, pattern specification, appendage development, neuron fate specification, neuromuscular processes and nervous system processes (Figure 7B). Furthermore, through the use of LASSO logistic regression analysis, we identified 19 characteristic genes including CALHM3, HRK, A4GNT, TMPRSS7, MAGEA10, TCF24, ZNF560, KCNG3, DLX6, SLC30A8, ZNF280A, DMRT2, SLC6A14, ISM2, CDK5R2, HMGA2, CDH10, NELL1 and OR14J1 (Figure 8A–S). Subsequently, a model score was calculated using the formula Model score = CALHM3 × 11.245 + A4GNT × 2.6103 + TMPRSS7 × 22.62 + MAGEA10 × 0.5965 + TCF24 × 9.1018 + ZNF560 × 2.2862 + KCNG3 × 21.903 + DLX6 × 0.169 + DMRT2 × 5.104 + SLC6A14 × 0.6867 + ISM2 × 3.0473 + CDK5R2 × 4.4217 + HMGA2 × 2.0624 + CDH10 × 5.3664. We observed that the model score robustly delineates risk groups (Figure 8T, ROC = 0.970).

## 4 DISCUSSION

Hepatocellular carcinoma, which makes up around 75%–85% of primary liver cancer incidences, remains the most prevalent variant.^23^ It's particularly prevalent in regions with high rates of hepatitis B or C infection, as these are key risk factors. Often detected at an advanced stage, HCC ranks as the third highest cause of cancer‐related mortality globally.^24^ Existing treatment options encompass surgical removal, liver transplantation, locoregional interventions and systemic therapies. Yet, the survival prospects continue to be unfavourable, primarily due to recurrent episodes and metastatic tendencies.^25^

In our study, we integrated the data from multiple independent HCC cohorts, including TCGA‐LIHC, ICGC‐FR and ICGC‐JP. Then, the enrichment score of 21 metabolism‐related pathways was quantified using the ssGSEA algorithm. Next, univariate Cox regression analysis was applied to identify the metabolic terms with significant correlation to patient survival. Finally, a prognosis model based on linoleic acid metabolism, sphingolipid metabolism and regulation of lipolysis in adipocytes was established, which showed good performance in predicting patient survival. Furthermore, we conducted a biological enrichment analysis to delineate the biological disparities between high‐ and low‐risk patients. Notably, we discerned differences in the microenvironments between these two patient groups. We also found that low‐risk patients could potentially respond better to immunotherapy. Drug sensitivity analysis suggested that low‐risk patients are more susceptible to bexarotene and erlotinib, yet exhibit resistance to ATRA and bleomycin. Furthermore, through the use of LASSO logistic regression analysis, we identified 19 characteristic genes, which could robustly indicate the risk groups.

Our results found that linoleic acid metabolism, sphingolipid metabolism and regulation of lipolysis in adipocytes were involved in HCC prognosis. Some previous studies have reported the role of these metabolic pathways in cancer. For example, Ogata et al. noticed that linoleic acid can lead to upregulation of miR‐494, further inducing cancer dormancy.^26^ Linoleic acid, an essential omega‐6 polyunsaturated fatty acid, has important roles in cell function and metabolism. Recent studies suggest it may be involved in the pathogenesis of HCC. Alterations in linoleic acid metabolism have been observed in HCC patients, potentially contributing to disease progression. Specifically, changes in enzymes involved in linoleic acid metabolic pathways may influence inflammatory responses, cell proliferation and cell survival, thus promoting tumorigenesis and progression.^27^, ^28^ However, the precise role of linoleic acid metabolism in HCC is complex and not fully understood, warranting further investigation for its potential as a therapeutic target or biomarker. For sphingolipid metabolism, Nagaraj et al. found that GOLM1 depletion can affect cell proliferation by regulating cellular sphingolipid metabolism.^29^

Biological enrichment analysis showed that the high‐risk patients might have a higher pathway activity of G2M checkpoints, KRAS signalling and E2F targets. The G2/M checkpoint, a critical control point in the cell cycle, ensures proper DNA replication before cell division.^30^ Dysregulation of this checkpoint can lead to uncontrolled cell proliferation, a hallmark of cancer.^31^ In many types of cancer, components of the G2/M checkpoint, such as cyclin‐dependent kinases (CDKs) and their regulatory proteins, are often overexpressed or mutated, leading to aberrant cell cycle progression.^32^ Strategies that target the G2/M checkpoint, such as CDK inhibitors, have been explored as potential cancer therapies. However, the effectiveness of these therapies can be influenced by various factors, including the genetic background of the tumour and the tumour's microenvironment.^33^ KRAS is an oncogene involved in cell signalling pathways that regulate proliferation, differentiation and survival.^34^ In HCC, KRAS mutations are rare but its pathway can be activated via other mechanisms, contributing to tumour development and progression.^35^, ^36^ E2F targets refer to genes regulated by the E2F family of transcription factors, critical for cell cycle regulation and DNA synthesis. In HCC, dysregulation of E2F targets can lead to uncontrolled cell cycle progression, contributing to tumorigenesis.^36^ Both KRAS signalling and E2F targets represent potential therapeutic strategies in HCC, with several drugs targeting these pathways under investigation. Understanding their roles can provide insights into the molecular mechanisms of HCC and improve the development of targeted therapies.

Our immune microenvironment analysis suggests that patients at high risk may exhibit an elevated infiltration level of Tregs. Tregs, constitute a subset of CD4+ T cells, performing essential immunosuppressive functions and playing a key role in sustaining immune homeostasis and tolerance. In the context of HCC, Tregs can be co‐opted by tumour cells to evade immune surveillance. Tregs tend to accumulate within the HCC tumour microenvironment, a phenomenon often associated with poor prognostic outcomes.^37^ By suppressing the effector T cells and promoting a tolerogenic environment, Tregs enable tumour progression. Targeting Tregs to relieve immunosuppression has emerged as a promising therapeutic strategy for HCC. However, a careful balance must be struck as excessive depletion of Tregs might trigger autoimmunity due to their role in maintaining immune tolerance.

## AUTHOR CONTRIBUTIONS

**Xijuan Tan:** Conceptualization (equal); data curation (equal); formal analysis (equal); software (equal); supervision (equal). **Sizong Chen:** Data curation (equal); formal analysis (equal); funding acquisition (equal); supervision (equal); validation (equal); visualization (equal). **Qiyi Luo:** Conceptualization (equal); formal analysis (equal); methodology (equal); resources (equal); software (equal); validation (equal). **Shenglin You:** Conceptualization (equal); funding acquisition (equal); project administration (equal); software (equal); supervision (equal); writing – original draft (equal). **Hankun Yuan:** Conceptualization (equal); formal analysis (equal); investigation (equal); resources (equal); software (equal); supervision (equal); validation (equal). **Jianchu Wang:** Conceptualization (equal); methodology (equal); project administration (equal); validation (equal); visualization (equal); writing – original draft (equal).

## FUNDING INFORMATION

This study was supported by the National Natural Science Foundation of China (No. 82060441 to JC Wang).

## CONFLICT OF INTEREST STATEMENT

None.

## Supporting information


Figure S1.

## Data Availability

All data are available from the corresponding author upon reasonable request.
