# Supplementary figures and images for: Identification of metabolism terms significantly affecting hepatocellular carcinoma immune microenvironment and immunotherapy response
Source: J Cell Mol Med. 2023 Nov 9;28(1):e18018. doi: 10.1111/jcmm.18018 (PMC10805494; doi:10.1111/jcmm.18018)

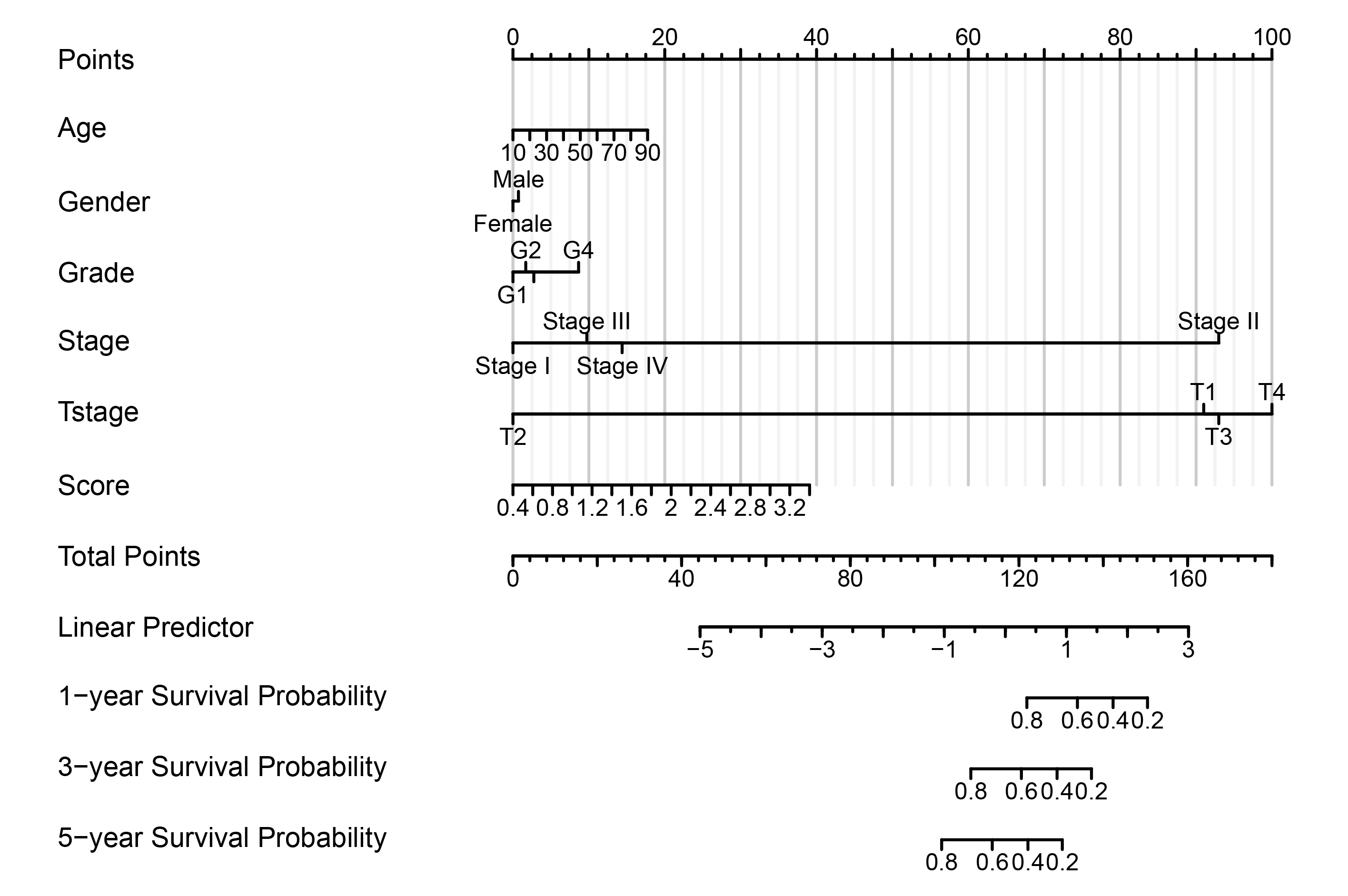

Supplement: Supplementary file 1 — Figure S1. [file JCMM-28-e18018-s001.tif]
